# Adrenal crises in adolescents and young adults

**DOI:** 10.1007/s12020-022-03070-3

**Published:** 2022-05-18

**Authors:** R. Louise Rushworth, Georgina L. Chrisp, Suzannah Bownes, David J. Torpy, Henrik Falhammar

**Affiliations:** 1 School of Medicine, Sydney, The University of Notre Dame, 160 Oxford St, Darlinghurst, NSW 2010 Australia; 2 Endocrine and Metabolic Unit, Royal Adelaide Hospital, Adelaide, SA Australia; 3 University of Adelaide, Adelaide, SA Australia; 4 Department of Endocrinology, Karolinska University Hospital, SE-17176 Stockholm, Sweden; 5 Department of Molecular Medicine and Surgery, Karolinska Institutet, SE-17176 Stockholm, Sweden

**Keywords:** Emergening adults, Adrenal insufficiency, Incidence, Mortality, Precipitating factors, Prevention

## Abstract

**Purpose:**

Review the literature concerning adrenal insufficiency (AI) and adrenal crisis (AC) in adolescents and young adults.

**Methods:**

Searches of PubMed identifying relevant reports up to March 2022.

**Results:**

AI is rare disorder that requires lifelong glucocorticoid replacement therapy and is associated with substantial morbidity and occasional mortality among adolescents and young adults. Aetiologies in this age group are more commonly congenital, with acquired causes, resulting from tumours in the hypothalamic-pituitary area and autoimmune adrenalitis among others, increasing with age. All patients with AI are at risk of AC, which have an estimated incidence of 6 to 8 ACs/100 patient years. Prevention of ACs includes use of educational interventions to achieve competency in dose escalation and parenteral glucocorticoid administration during times of physiological stress, such as an intercurrent infection. While the incidence of AI/AC in young children and adults has been documented, there are few studies focussed on the AC occurrence in adolescents and young adults with AI. This is despite the range of developmental, psychosocial, and structural changes that can interfere with chronic disease management during this important period of growth and development.

**Conclusion:**

In this review, we examine the current state of knowledge of AC epidemiology in emerging adults; examine the causes of ACs in this age group; and suggest areas for further investigation that are aimed at reducing the incidence and health impact of ACs in these patients.

## Introduction

Adrenal insufficiency (AI) is a rare disorder which has an estimated prevalence of 120/million in childhood, increasing to 300/million in the adult years [1, 2]. Aetiologies vary according to age but are predominantly congenital in early life, with congenital adrenal hyperplasia (CAH) being the commonest cause of primary AI (PAI) among children and adolescents (incidence between 1/14,000 and 1/18,000) [2, 3]. Malignancies and autoimmune conditions, among other aetiologies, emerge in the later years of childhood and into adulthood [2–4]. Secondary adrenal insufficiency (SAI), typically resulting from congenital anomalies, tumours in the hypothalamic-pituitary area and trauma, occurs more rarely than PAI in children and younger people (15–40% of all cases) [2, 3, 5]. Glucocorticoid-induced AI is thought to be relatively common, frequently undiagnosed, and can occur at any age, as can other iatrogenic forms of AI arising from, for example, treatment of cerebral tumours (irradiation or surgery) or pharmacotherapies, such as immunotherapy for certain malignancies [6, 7]. Symptoms of AI are often non-specific and insidious and include fatigue, unexplained weight loss, anorexia and nausea, postural dizziness, and hyperpigmentation (in PAI). Electrolyte abnormalities (hyponatraemia, hyperkalaemia) may also be present, especially in PAI [1, 8]. In a recent study 84% of patients with PAI had hyponatraemia and one third hyperkalaemia at the time of diagnosis [9]. Delays in identification of AI symptomatology may lead to development of an adrenal crisis (AC), which is a life-threatening episode of AI, characterised by hypotension, electrolyte abnormalities, hypoglycaemia (in children), alterations in consciousness and acute abdominal symptoms [2, 8, 10]. Rarely young patients with AI may be diagnosed at presentation in an AC, often after multiple clinic visits with symptoms suggestive of AI that were unrecognised, and tragically these may be fatal [11, 12].

All patients with AI require daily or more frequent glucocorticoid replacement therapy, with a mineralocorticoid prescribed for those with PAI. During periods of stress, all patients are at risk of an AC, estimated incidence of 6 to 8 ACs/100 patient years [8, 13]. For this reason, competency in self-management of glucocorticoid replacement therapy, including dose escalation (oral and/or parenteral) at times of physiological stress, is essential in all age groups [1, 8, 10, 14]. For adolescent and young adult patients, however, attainment of skills in AI self-management is just one of the complex developmental tasks to be confronted during this period. It is likely, therefore, that some patients in this age group experience difficulties adjusting to the demands of living with a chronic lifelong disorder and that this may be associated with an increase in AC incidence during this time [15].

Despite its importance, information on the epidemiology, causes and possibilities for prevention of AC events in emerging adults is scarce. In this review, we examine the current state of knowledge about the epidemiology of ACs in adolescence and early adulthood; review the likely underlying precipitants of ACs in this age group; and suggest areas for further study that are aimed at reducing the incidence and consequent health impact of ACs in these patients.

## AC incidence, morbidity, and mortality

No systematic investigations into AC incidence and risk factors among adolescents and young adults have been conducted. Rather, AC epidemiology in emerging adults is assumed to be comparable to that among adults generally. Research that does include information on ACs in this group is typically aimed at determining AC rates in children or in the general adult populations, with AC rates among emerging adults reported incidentally. More commonly, however, AC incidence among adolescents and young adults is not investigated or identified in this context.

Several case reports of ACs in emerging adults have been published [16–21]. These illustrate problems in diagnosis and treatment and are instructive but cannot provide incidence estimates. Population-based studies, by comparison, have the potential to provide unbiased estimates of AC rates (see Table 1). One such study, which used data on hospital admissions among patients with a principal diagnosis of an AC from a national repository, investigated AC rates by age group for all forms of AI, and reported an average annual AC admission rate of 7.2/million in the 15–19 years age group [22]. This corresponded to a rate of approximately 6 AC admissions/100py among the estimated AI patient population [22] (Table 1). As only principal diagnoses of an AC were included, these approximate admission rates would be an underestimate of the true AC admission rate. A similar study, among, adults using state-wide hospitalisation data that included all admission diagnoses, found an average AC admission rate of 8.3/million/year among 20–29-year-old patients [23], although the rate in the 20–24 year age group was not identified explicitly.

**Table 1:** Adrenal crisis incidence in adolescents and younger adults

Study type and author (reference)	Year	Country	Age (yrs)	Design/sample	AC incidence (by age group)	
					*Adolescent*	*Early Adult*
Retrospective study of AC in childhood						
Chrisp et al. [24]	2018	Australia	0–18	Audit/CAH	4 ACs (6.9% admissions)^b^	–
Rushworth et al. [27]	2021	Australia	0–18	Audit/SAI	–	–
Eyal et al. [30]	2019	Israel	0–18	Audit/All AI	–	–
Population-based (children)						
Rushworth et al. [22]	2017	Australia	0–18	National data/All AI	7.2/million/yr (15–19 yrs)^a^	–
Rushworth et al. [25]	2016	Australia	0–18	Hospital data/CAH	5 ACs (5.7% admissions)	–
Population-based (adults)						
Rushworth et al. [23]	2014	Australia	18+	Admission data/all AI	–	8.3/million/yr (20–29 yrs)
Retrospective study of AC/AI in adulthood						
Reisch et al.^a^ [27]	2012	Germany	18+	Mixed/incl record review/CAH	–	
Goubar et al. [28]	2019	Australia	18+	Audit/hospital records/PAI	–	40% of 41 admissions^c^
Omori et al. [26]	2003	Japan		All AI (majority SAI)	–	4 ACs in SAI patients only

*AC* adrenal crisis, *AI* adrenal insufficiency, *CAH* congenital adrenal hyperplasia, *SAI* secondary adrenal insufficiency, *PAI* primary adrenal insufficiency

^a^Principal diagnosis of an AC only

^b^10–18 years old

^c^Approximate, 20 to 29 years

A small number of studies have provided additional information on AC epidemiology in this age group [24–30] and, of these, one longitudinal study of patients with CAH in Germany demonstrated the value of separate identification of AC incidence in emerging adults. It reported, that, from early childhood, there was a general decrease in AC events with age, but that there was a paradoxical increase in AC episodes among patients aged 18–25 years, followed by a continuation of the overall decline [27]. Although the incidence rate was not calculated, a retrospective study of hospitalisations in adult patients with PAI in Australia found that diagnosed ACs comprised a considerable proportion (38.9%) of acute medical admissions for patients aged 20 to 29 years with AI [28]. In contrast, a study of adult patients with AI from Japan identified only 4 ACs in this age group, all of whom had SAI [26]. As ACs are slightly more common in primary than secondary AI, this result is somewhat unexpected, but may be related to the presence of comorbid diabetes insipidus (DI), which is associated with an increased AC risk [31, 32].

Mortality is known to be increased in patients with AI, with deaths from an AC and infection being well-documented causes [33–35]. However, AI-related mortality in the emerging adult group has not been studied specifically. Case reports, often after coronial investigations, provide salutary evidence of deaths due to ACs in young people, frequently in situations where AI was not recognised for some time, or not at all, by health professionals [11, 12]. While these case reports are important and of educational benefit to clinicians, they cannot provide reliable information on mortality rates.

### Epidemiological considerations

Improving outcomes for patients with AI requires ongoing access to reliable AI/AC incidence data. Problems with access to AC-related information in emerging adults have many features in common with those encountered in other age groups. Among these, is the rarity of AI and its subtypes, and the even greater rarity of ACs, so that it is difficult to amass unbiased patient samples for research purposes. Further, the ongoing absence of a universally accepted and applied definition of an AC, means that any variability in incidence estimates, over time or between different sub-populations, is difficult to interpret with any confidence [8, 36].

All data sources and study designs have inherent strengths and weaknesses. Large, routinely collected databases can provide incidence measurements for rare disorders, such as AI. Typically, these are unmatched, routinely collected datasets, such as hospital admission data, which do not differentiate individual patients from pooled admissions and cannot identify factors associated with high AC frequency in some patients. These data rely on coding of diagnoses made by the attending clinician, which may be affected by classification bias (especially with regards to the definition of ACs), coding errors, and missing information [36]. By comparison, cohort studies vary in the way that they enumerate events (whether through self-report or documented episodes of illness that are reviewed by clinicians, or a combination of these) and in the criteria that are used to differentiate an AC from a milder episode of illness due to AI. Surveys of support groups, on the other hand, offer access to detailed information from participating members but are typically affected by important biases that influence adversely the validity and generalisability of the results.

Other issues affect research into the emerging adult age group specifically. Among these, is the problem of collecting data from the, often separate, specialist paediatric and general adult health systems. Cohort studies, based initially in a paediatric setting, may be affected by “loss to follow-up” of patients when they move into the adult system. Matched data analysis, using the medical record identifier only, may be unable to access data from the two systems, as identification numbers may differ between hospitals and services. Direct patient surveys can be affected by selection biases, due to accessibility to some patients and inaccessibility to others, who may be lost to the health system and unconnected to AI support networks.

While limitations in access to reliable data are considerable in this age group, indirect evidence on effectiveness of preventive interventions may be obtained from secondary data sources. One study used data on medical jewellery uptake in patients aged up to 24 years and found that subscriptions diminished among emerging adults, reflecting clinical experience which indicates that adherence to AC prevention recommendations diminishes in this age group [37].

Data on deaths from an AC are another important outcome indicator. Deaths among patients with known AI should be differentiated from those without a pre-existing AI diagnosis, as each represents one or more failures to identify and manage AI across the spectrum of disease and should be investigated [36]. Monitoring mortality without access to medical details and using death certification alone is likely to underestimate the contribution of AI/AC to the mortality rate, as AI may be unrecognised as the cause of death or may be omitted as a comorbid condition on the death certificate [38]. Coronial records are another source of information of AC-related deaths in emerging adults but these are rare, and the findings and recommendations are frequently delayed and may not be widely disseminated [11].

### Definitional issues, clinical factors, and treatment

#### Definition

Definitional issues are an ongoing problem in AI/AC prevention research. Despite attempts to address this problem, AC definitions differ between studies and clinicians. Although opinions on precise elements of an AC definition may vary, there is general agreement that use of consistent definitions that have sufficient diagnostic specificity to distinguish an AC from milder illness (symptomatic AI) is important. While an AC diagnosis is highly relevant to the management of an individual patient and should prompt evaluation of each patient’s self-management skills and other aspects of their care, misclassification of AC/symptomatic AI is especially problematic with regards to assessment and monitoring of trends and variations in AC incidence [36]. To this end, definitions of an AC have been proposed for use in both adults and children [2, 8]. A recommended definition for adults states that: “An AC is an acute deterioration in health status associated with hypotension (absolute or relative) with features that resolve within 1 to 2 h after parenteral glucocorticoid administration (ie a marked resolution of hypotension within 1 h and improvement in clinical symptoms over a period of 2 h). Other concomitant symptoms and signs of an AC include biochemical abnormalities: hyponatraemia, hyperkalaemia, hypercalcaemia, hypoglycaemia (usually in children). Suggestive symptoms include: nausea; vomiting; pain (abdominal, limb, back); severe fatigue; severe weakness; postural dizziness/syncope; and confusion. Consideration of the effects of incidental illness as causes of the major features, in particular shock, improves the specificity of diagnosis” [11].

This definition would be appropriate for most adolescents and young adults but there may be younger adolescents for whom an alternative characterisation of haemodynamic disturbance (hypotension or sinus tachycardia relative to age-related normal levels) may be a better measurement [2].

#### Clinical factors

While strategies for the management of AI and prevention of ACs are common across the age spectrum, there are some factors in the emerging adult group which may make management more challenging. Among these, delays in the diagnosis of AI, which may result in presentation in an AC, occur in all age groups but clinical experience and case reports suggest that this may be an important feature of AI/AC epidemiology in emerging adults, as symptoms of evolving AI overlap with those of other relatively common disorders, such as weight loss due to an eating disorder, lassitude following a viral illness, and depressive disorders, all of which occur in this age group [11, 18, 39].

Missed or delayed replacement doses can reduce well-being and act as precipitants of an AC [8]. Non-adherence to replacement therapy occurs in AI [40] and may be more common among emerging adults for a variety of reasons, such as financial and psychosocial factors, including housing insecurity, diminished access to medical care, and drug and alcohol usage. Regimens which rely on the use of split doses are more demanding and are also more likely to be forgotten. Social pressures in adolescence and early adulthood may result in denial or suppression of information about a patient’s diagnosis and may also lead to use of smaller or omitted replacement doses, against medical advice, to avoid or reduce weight gain, particularly among females. Good treatment adherence, on the other hand, has been associated with a better quality of life in patients with AI [41].

Physiological factors may also play a role in an increase in AC risk in this age group. Adolescence is a period of physiological transformation characterised by rapid growth and changes in the hormonal milieu [15]. Sex hormone as well as growth hormone and IGF-1 production are increased during puberty. Simultaneously, 11β-hydroxysteroid dehydrogenase type 1 enzyme activity is decreased due to inhibition from increased IGF-1 levels [42]. A rise in circulating thyroxine concentrations, from thyroxine administration, which may be needed for concomitant hypothyroidism of primary or secondary aetiology, or an increase in thyroxine production in comorbid thyrotoxicosis, induces the 11β-hydroxysteroid dehydrogenase type 2 enzyme, thereby increasing hydrocortisone metabolism, which can increase AC risk in patients with AI [43].

As a consequence of the physiological changes occurring during puberty, higher doses and, at times, more frequent dosing of hydrocortisone is needed [44]. However, in order not to impair growth, doses more than 17 mg/m^2^/day should not be used [45]. More usual doses for cortisol replacement in children are ~8 mg/m^2^/d, in divided doses, with adjustment for individual need, taking into account marked differences in cortisol metabolism and individual factors [1]. Thus, doses of glucocorticoid replacement may need regular review and modification to address the physiological demands of rapid growth and puberty.

#### Recommendations for treatment of AI/AC

Treatment of an AC involves urgent administration of hydrocortisone (IV or IM if IV access is unavailable) and fluid resuscitation [8, 10]. Further details on treatment of adults and children with AC can be viewed in Table 2.

**Table 2:** Recommended treatment of an adrenal crisis

Age group	Hydrocortisone	Fluids	Additional measures (if relevant)
Adults	Prompt administration of 100 mg IV (or IM)	IV 1000 ml of normal saline (0.9% isotonic sodium chloride) in the first hour	Antibiotic therapy, admission to intensive care or high-dependency unit, administration of low dose heparin
	Follow with 200 mg every 24 h (continuous infusion or IV/IM boluses (50 mg) every 6 h	Add IV dextrose to 5% concentration in normal saline, if hypoglycaemic	
	If initial treatment is successful (usually after 24 h), oral hydrocortisone at 2 to 3 times the usual dose, tapering to the usual dose over the next 2 to 3 days^a^	Then administer crystalloid fluids according to standard resuscitation guidelines^b^	
Adolescents and young adults	Please use the recommendations for adults or children depending on age and development stage		
Children	Prompt administration hydrocortisone at 50 mg per square metre of body-surface area IV (or IM), followed by 50–100 mg per square metre every 24 h, given as a continuous infusion or IV (or IM) boluses (12.5–25 mg per square metre) every 6 h	Bolus of normal saline at a dose of 20 ml per kilogram of body weight, with repeated doses up to a maximum of 60 ml per kilogram in the first hour, along with intravenous dextrose, 0.5–1 g per kilogram, if hypoglycaemic	
	If initial treatment is successful (usually after 24 h), oral hydrocortisone at 2 to 3 times the usual dose, tapering down to the usual dose over the next 2 to 3 days^a^	Then administer crystalloid fluids according to standard resuscitation guidelines^b^	

Adapted from ref. [8]

Extra Information:

Prompt investigation of other causes when hypotension persists despite adequate initial treatment. Precipitating events (e.g. sepsis, gastroenteritis) should be considered. If hydrocortisone is unavailable, another parenteral glucocorticoid, such as dexamethasone (4 mg every 24 h), methylprednisolone (40 mg every 24 h), or prednisolone (25 mg bolus followed by two 25 mg doses, for a total of 75 mg in the first 24 h; thereafter, 50 mg every 24 h), may be used

^a^Fludrocortisone replacement is not required if hydrocortisone doses exceed 50 mg every 24 h but is typically administered in adults and children with primary adrenal insufficiency when oral hydrocortisone is started

^b^Circulatory status, body weight, and relevant coexisting conditions should be taken into account

### Risk factors

Psychosocial issues per se have not been documented as risk factors for an AC although “stress” is a frequently reported precipitant [13]. Psychosocial difficulties are commonly encountered in the adolescent and young adult years, varying according to age, developmental stage and between individuals. While it is unlikely that longer term, more pervasive stress would act as a precipitant of an acute episode of AI, it is probable that chronic stress experienced by emerging adults would detract from achieving optimal AI self-management. Clinical experience suggests that it is possible that stress arising from issues related to insecure housing, financial problems and poor access to healthcare would increase the likelihood of missed replacement doses and lower the prospects for appropriate use of emergency measures in the context of intercurrent illness. It should be noted though that no studies were found to back these theories, and these were based on clinical experience and common sense. Intermittent or frequent use of drugs and alcohol, which may co-occur with psychosocial difficulties, could increase the probability of irregular dosages (late or missed doses) of replacement therapy and delayed or absent institution of emergent measures for an impending AC. Common sequelae of drug and alcohol exposure, such as nausea and vomiting, increase the risk of poor absorption of replacement therapy, which, in turn, would substantially increase AC risk.

Late adolescence and early adulthood are also life stages typically associated with changes of residence, healthcare providers (including changes to health insurance in some countries) and work or study. Access to clinical expertise may depend on a patient’s place of residence, their ability to access care during the working week, and financial status. These factors, together with difficulties in navigating the transition from paediatric to adult services, mean that some patients lose contact with the health system following discharge from the paediatric health service and may be at increased AC risk, due to the combination of poor regular care and fewer opportunities for educational reinforcement of preventive strategies. The extent of this problem is unknown but there is evidence indicating that up to half of the young adults with an endocrine disorder are lost to follow-up after transfer to adult healthcare services [46].

Other factors, such as the presence of comorbid illnesses, are less related to a specific life stage but are nevertheless associated with increased AC risk. Among these are comorbid conditions such as diabetes insipidus (DI), which is relatively common among patients who are survivors of cerebral tumours and some congenital forms of AI [31, 32]. Similarly, patients with comorbid diabetes mellitus (DM) may be at increased AC risk, possibly due to the complexities of managing an intercurrent illness in the presence of both disorders, although the evidence supporting this is variable [47–49]. A decreased requirement for insulin in an emerging adult with type 1 diabetes may be an indicator of AI [50].

Although not widely investigated, there is some evidence to indicate that gender is associated with variation in AC incidence in this age group. One study of sex differences in admissions for AI/AC in childhood in Australia found that, among 10–14-year olds, males had significantly higher AC rates than females [22]. There were also higher rates of admissions for secondary AI among males in the 10–14 and 15–19 year age groups. While the reasons for these differences are not certain, cerebral tumours and traumatic brain injuries, which are associated with both SAI and comorbid DI, are known to be more common in young males [51].

Evidence indicates that, in the general population of patients, some individuals appear to be at increased risk of an AC, having frequent episodes, while others may be observed for many years without an AC [31]. Patients with primary AI are considered to have a slightly higher risk of an AC than those with secondary AI due to some preservation of cortisol secretion in SAI [8]. Physiological/genetic factors may also play a role in AC susceptibility. For example, a considerable proportion of patients with AI in the emerging adult group have CAH and those with the severe form of enzyme deficiency (null or In2G genotype) have been shown to have a higher AC risk than those with less severe abnormalities [27]. This difference could be due to the more severe aldosterone deficiency and may also be related to epinephrine insufficiency in this group [52].

Iatrogenic factors may also inadvertently increase AC risk in some individuals. Recent recommendations for replacement therapy aim to reduce the adverse effects of glucocorticoid exposure by attempting to recapitulate physiological cortisol levels through the use of divided doses (two or more per day) of short-acting glucocorticoids (hydrocortisone or cortisone acetate) [1]. However, this may be associated with periods of profound hypocortisolaemia which may increase AC susceptibility in some patients [53–55]. Delayed or sustained release formulations that are available in some countries may ameliorate this risk [10, 44]. In addition, therapeutic glucocorticoid exposure for treatment of a range of problems in adolescence and early adulthood may induce iatrogenic AI, which may be unrecognised until a patient presents in an AC [56]. A number of other medications, including opiates, have been associated with the development of AI and ACs, and these may also be relevant in this age group [57]. Immunotherapy with immune checkpoint inhibitors for melanoma and other malignancies have also been associated with the development of AI and AC [58], however, these are less commonly used in this age group.

## Precipitating factors

Intercurrent infection is the most often cited precipitant of an AC in treated AI [8, 13]. Viral infections predominate in children, while bacterial infections are much more common in older adults [14, 23, 24, 59]. Gastroenteritis is frequently identified as an AC precipitant, especially in children and young adults [2], but this association may be overestimated, as it is difficult to differentiate between typical symptoms of gastroenteritis and the acute abdominal symptoms of an AC. Nausea and vomiting, which may be mistaken for gastroenteritis, are also side effects of excess alcohol intake and other forms of recreational drug use, and their presence may result in missed or late doses of hydrocortisone or inadequate absorption of replacement therapy in the emerging adult group. An AC may occur in this context because oral stress dosing is unlikely to be effective and patients may be unaware or unable to use recommended parenteral doses of hydrocortisone, or they may not have access to these in a domiciliary setting.

It is likely that the relative frequencies of precipitants such as infections differ in adolescents and young adults from those in other age groups. In this population, viral infections, particularly those with gastrointestinal symptoms, are likely to be the most common infective cause. Other causes, such as accidents and injuries may also be relatively common precipitants in this age group, particularly among males [51]. Missed doses or non-adherence to recommended replacement schedules are likely to be more common among emerging adults than children and older people. As has been mentioned, drug and alcohol exposure can precipitate an AC through missed, delayed or non-absorbed replacement therapy.

## Prevention

**Fig. 1:**
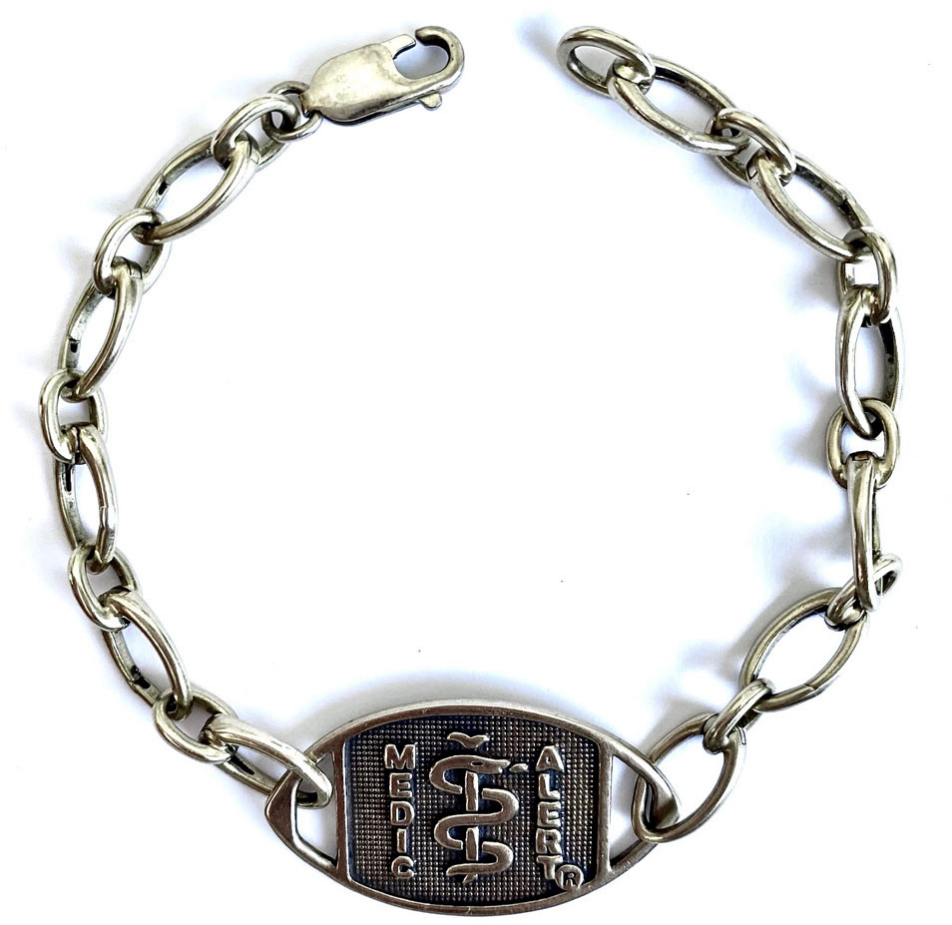
Medical Jewellery with emblem

Education of patients (and their parents/associates) to achieve competence in AI self-management is regarded as an essential component of AC prevention in all age groups [1, 8, 13]. To prevent an AC, all patients need to be provided with injectable glucocorticoids and appropriate equipment so that they can inject themselves (or receive an injection from a relative/associate) during periods of physiological stress or in circumstances where oral treatment cannot be taken or absorbed or has not been effective [8]. In addition, all patients with AI should carry identification noting their steroid dependence and use of medical jewellery is recommended [2, 8, 14] (Fig. 1). Continual educational reinforcement about the importance of adherence to recommended replacement schedules and the appropriate use of both oral and parenteral stress dosing is advised for all patients [1, 8, 13, 60]. However, education alone does not appear to be sufficient for many patients and evidence suggests that direct experience of an episode of acute AI/AC improves competency, especially with regards to use of stress doses [13, 24, 27, 28, 32].

Risks of poor management/adherence during the transition (paediatric to adult care) period are likely to vary between patients and may be greater among less advantaged groups but there is a paucity of information on evidence-based strategies to improve AI treatment adherence in this age group. Given this, evidence from studies on transition among young people with other chronic health problems, such as diabetes mellitus, can potentially inform service provision for AI patients [61, 62]. Results from such investigations indicate that interruptions to the smooth transition from the paediatric to adult health services are pervasive and specific interventions directed at this area, taking into account the personal and social context of each patient, should improve adherence [61, 63, 64]. An absence of effective transition may contribute to healthcare fragmentation, decreased frequency of clinical follow-up, and adverse outcomes, with risk factors including loss of health insurance coverage, increased risk-taking behaviours, and difficulty coping with added responsibilities [61, 65]. To address potential problems, gradual transition in well-educated and prepared patients is recommended [15, 64, 66].

Other factors that may affect AC prevention in emerging adults involve the need for managed sequential handover of responsibility of AI/AC prevention from parents/carers as the young person moves from adolescence to adulthood. This may be a source of stress for both carer and patient, particularly among patients who have had AI since early childhood or who have had difficulties managing their disease. A fairly common cause of SAI in children which later become adolescents and young adults is craniopharyngioma and other cerebral tumours in the hypothalamic-pituitary region. Both the tumour per se and treatment (i.e. surgery and radiotherapy), which does not only affect the pituitary function but often also may affect cognitive function. The impact of mild cognitive impairment may affect transition into adulthood and patients’ own responsibility of medications and adherence to sick-rules. Parents and carers may need education and support to assist them with managing this paradigm shift in AI management. Assessment of transition readiness together with checklists to detect areas for intervention are a sensible and practical recommendation [64]. With regards to AI, awareness of the life-threatening nature of an AC and the understanding that there is a requirement to take responsibility for its prevention may induce anxiety in some patients, and this may manifest paradoxically in neglect of warning signs and symptoms and hesitancy in taking appropriate interventions to prevent an AC.

Tailored interventions to assist the development of self-management skills are also of great potential benefit in this age group [67]. Electronic web-based prompts and educational programs (for example: those from https://adrenals.eu/about/what-is-adrenalnet-mission/) may be especially valuable and acceptable among emerging adults. Silent alarms on electronic equipment, such as smart watches, may be useful devices to remind patients to take intraday doses, without causing embarrassment. Peer support can be of value to some patients, particularly in this age group but it may be difficult to initiate given the rarity of this disorder.

## Future research directions

Adrenal crisis incidence, risk factors and the most effective preventative strategies are all poorly described in the emerging adult age group. As a priority, systems to evaluate AC/AI incidence and mortality in patients in this age group should be established and subjected to regular review. In addition, more detailed investigations, examining AC risk factors among emerging adults should be conducted. New metrics to assess successful transition into the adult health system should be assessed and regularly reviewed.

Research into new treatments, such as cell- and gene-based therapies and other regenerative medicine approaches [68, 69], if effective, would assist in ameliorating AC risk by lessening or removing the patient’s reliance on glucocorticoid replacement therapy. Early human trials of insertion of the *CYP21A* gene into the genome through adenovirus transfection are underway, offering potential for long term amelioration of cortisol and aldosterone deficiency and relief of hyperandrogenism in patients with the commonest form of CAH, 21-hydroxylase deficiency, the commonest cause of AI young people (https://adrenastx.com/). Adrenal cell transplants have been used in animal studies but have not yet been used in a clinical trial [70]. Auto-injectors to facilitate prompt IM hydrocortisone at home are under development (https://solutionmedllc.com/#).

## Conclusions

Emerging adulthood represents an under-researched but important period in the life of patients with AI. Although the extant evidence is sparse, it appears that AC risk increases during this time, which is consistent with experience in the health course of adolescents and young adults with other chronic disorders. Multiple factors increase the vulnerability to poor outcomes in adolescence and early adulthood. These include difficulties in managing the transition from paediatric to adult health services; poor adherence to glucocorticoid replacement regimens; problems with decision making regarding the use of glucocorticoid dose escalation independently of parents; and broader psychosocial and economic issues related to living with a chronic illness. To address this increase in morbidity from AI, there is a need for better access to AC incidence data according to age, in addition to more detailed research investigating the relationship between the multiple physiological, personal and social factors that feature in this period of life and AC risk.
